# Ohio physician attitudes toward medical Cannabis and Ohio’s medical marijuana program

**DOI:** 10.1186/s42238-020-00025-1

**Published:** 2020-04-21

**Authors:** Emilia Lombardi, Joshua Gunter, Erin Tanner

**Affiliations:** Department of Public Health and Prevention Sciences, Baldwin Wallace University, 275 Eastland Rd, Berea, OH 44017 USA

**Keywords:** Ohio, Medical marijuana, Medical Cannabis, Cannabis attitudes and beliefs, Physicians

## Abstract

**Background:**

Ohio’s medical cannabis program is one of three states that require physicians to become certified to recommend medical cannabis to their patients. The current study examines the attitudes of Ohio physicians toward medical cannabis and Ohio’s program to ascertain how likely physicians are to participate in Ohio’s program.

**Methods:**

Physicians were invited to complete an internet survey that asked them about their concerns regarding medical marijuana, Ohio’s program, their likelihood of recommending medical cannabis, and becoming certified within the state. Ordinal and logistic regressions were used to understand the physicians’ likelihood of recommending cannabis, of becoming certified to recommend cannabis, and their attitude toward Ohio’s program.

**Results:**

In total, 11,665 physicians licensed to practice in Ohio were contacted by email, and 344 responses were received for a response rate of 2.9%. Only 42 physicians reported being certified or had plans to become certified to recommend marijuana, and 62% were unlikely to recommend marijuana to their patients. Overall, the belief that medical cannabis should be legal had the greatest association with the likelihood of recommending cannabis (OR = .37, 95% CI = .24–.54), of becoming certified (OR = .21, 95% CI = .10–.38), and believing that Ohio’s program is too strict (OR = .39, 95% CI = .30–.51). However, the study sample precludes generalizing the results beyond this study. The 2.9% response rate could indicate a bias toward physicians who have strong opinions about the legality of medical cannabis.

**Conclusion:**

The results show that many physicians have concerns about medical cannabis and Ohio’s program, and many physicians may not participate in the program. This could be a problem for patients who would like to use cannabis for medical reasons; therefore, these patients, may need to utilize one physician for cannabis and another for regular care. Physicians will likely be caring for patients who are using cannabis regardless of their own beliefs about it. The lack of training regarding cannabis in healthcare, along with requiring “certified recommenders” to have training could result in a fractured healthcare system.

## Background

Medical cannabis became available to Ohio residents September 2018 (131st Ohio General Assembly, 2016). The state has created a system to allow people access to medical cannabis after previous voter referendums had failed. The state program requires physicians to become certified in order to recommend cannabis and limits the use of cannabis to 21 conditions. From 2016 to 2019 the Ohio Department of Commerce received and approved permits from cultivators, processors, and retailers, but questions remain regarding physician participation. Physicians who are interested in recommending cannabis to their patients had to participate in a two-hour training that encompassed cannabis safety and treating qualified conditions. Physicians also require a medical license without any prohibitions or restrictions.

While the general public’s attitude toward cannabis has become more positive, the attitudes of physicians may not be changing as quickly (McCarthy 2018) This can have a significant impact in states like Ohio where physicians must become certified to recommend cannabis to their patients. Little is known about the factors physicians use in deciding to participate in medical cannabis programs. Physicians receive little training in medical school on the topic and may not have sufficient knowledge on which to base their decisions (Evanoff, et al. 2017).

An earlier study of physician attitudes about medical cannabis found that a minority supported the provision of cannabis to patients (Charuvastra et al. 2005). Over time more physicians became supportive of medical cannabis, but that support varied from study to study. One study in Colorado found a minority of physicians was willing to recommend medical cannabis and believed that it could provide medical benefits (Kondrad and Reid 2013). A study with New York physicians (primarily New York City) found that most believed patients should be able to access cannabis and were willing to refer patients to doctors that are certified to recommend cannabis (Sideris et al. 2018). While studies have shown there is varying physician support for medical cannabis, they have also found that physician knowledge was limited (Brooks et al. 2017; Braun et al. 2018; Philpot et al. 2019).

Now that Ohio has established a medical cannabis program that requires physicians to become certified in order to participate, the question of what makes physicians willing to recommend cannabis and become certified is increasingly important in understanding the success of the program. The State Medical Board of Ohio conducted its own survey of physicians in 2016 and found most physicians are unlikely to recommend cannabis (State Medical Board of Ohio 2016). They asked physicians what would increase their likelihood of recommending cannabis, and approximately 65% stated peer reviewed research and 45% stated more training and education. It is not generally known whether physicians would seek certification.

Requiring physicians to be certified in order to recommend cannabis exists in other states (Massachusetts and New York) and could be used in the future by other states who seek to create their own medical cannabis program. However, physicians may not seek certification, and this can limit the effectiveness of the program. As in New York, patients in Ohio were not be able to access cannabis for a time due to the limited number of certified physicians (Drug Policy Alliance 2016; Lewis 2016). Physicians’ willingness to become certified to recommend cannabis is important for the success of the program. This study examined the association between physician beliefs and attitudes, and the following factors:

1. Their likelihood of recommending cannabis to their patients.
2. Their interest in becoming certified in the state to recommend cannabis
3. Whether they believed that Ohio’s program was too strict or too lenient.

The results will provide a greater understanding about Ohio’s program an in its future. It will also provide an understanding of the factors that may lead physicians to become certified. This information would be beneficial for other states that may consider similar policies for their medical cannabis programs.

## Methods

The study utilized the word marijuana rather than cannabis in order to mirror the language in Ohio’s Law. Ohio physicians were recruited through the Ohio Board of Physicians roster to participate in an internet-based survey that investigated their opinions about medical marijuana and Ohio’s new program (State Medical Board of Ohio, 2018). We emailed 11,665 physicians between May 2018 and the end of July 2018 asking for their participation in a study examining their attitudes and beliefs about medical marijuana and Ohio’s program. We received 344 responses in total for a response rate of 2.9%, but listwise deletion of missing data reduced the total to 314 (274 for examining attitudes toward Ohio’s program).

### Dependent variables

The three questions asked in the survey were 1) How likely would you be, in general, to recommend medical marijuana to patients, 2) What is your opinion about Ohio’s medical marijuana program, 3) How interested are you in becoming certified to prescribe marijuana in Ohio. The likelihood to recommend marijuana used a five-point scale that ranged from extremely unlikely, somewhat unlikely, neither likely nor unlikely, somewhat likely, extremely likely to recommend medical marijuana to a patient. The variable was then collapsed into three categories, unlikely (− 1), neither likely nor unlikely (0), likely (1). The physicians’ expressed their opinion about Ohio’s program as being a) too lenient (− 1), b) just right (0), c) too strict (1). The physicians indicated their interest in becoming certified to recommend marijuana by identifying that they were a) already certified or had plans to seek certification (1), or b). had no plans to seek certification (0).

### Independent variables: physicians’ beliefs about medical marijuana

The study examined physicians’ beliefs about medical marijuana (utility and safety) by asking them to respond to the following seven statements:

- Medical marijuana should be legal (high score indicates negative belief about marijuana)
- Medical marijuana is safe when used responsibly (high score indicates negative belief about marijuana)
- Marijuana has medicinal uses (high score indicates negative belief about marijuana)
- Legalizing medical marijuana would cause crime rates to increase (high score indicates positive belief about marijuana)
- Medical marijuana will hurt the war on drugs (high score indicates positive belief about marijuana)
- Marijuana is harmful to people (high score indicates positive belief about marijuana)
- Those supporting medical marijuana is primarily interested in recreational use. (high score indicates positive belief about marijuana)

The responses to all seven questions consisted of strongly agree, somewhat agree, neither agree nor disagree, somewhat disagree, or strongly disagree, and raged from one to five.

### Independent variables: concerns about medical marijuana

Physicians were asked about their concerns about medical marijuana. On a four-point scale (not concerned to extremely concerned), physicians indicated that they were concerned about medical marijuana’s

- safety,
- consistency of quality,
- concern about federal laws
- psychoactive effects,
- limited evidence of therapeutic benefits, and
- potential addiction.

### Independent variables: perceived knowledge and ability

Physicians were asked about their own perceived knowledge and ability to answer patient questions about medical marijuana. Physicians were asked if they strongly agree (1), somewhat agree (2), neither agree nor disagree (3), somewhat disagree (4), strongly disagree (5). whether they considered themselves:

- knowledgeable about medical marijuana,
- comfortable answering patient questions about

- marijuana efficacy,
- safety, or
- drug interactions.

### Analysis

Analysis was conducted using R v3.5.2 and Rstudio v 1.2.1335, along with Tidyverse, pastecs, and RQDA packages (RStudio Team 2016; Wickham 2017; Grosjean and Ibanez 2018; Huang 2018; R Core Team 2018). Descriptive statistics were first assessed using base R statistics to identify percentages, means, and standard deviations. Ordinal and logistic regression was used to examine the likelihood of recommending medical marijuana and the opinions of Ohio’s medical marijuana program controlling for other measures. Odds ratios and 95% confidence intervals were reported for all variables within the analyses.

Likelihood to recommend marijuana to patients, and physician opinion about Ohio’s program are both polytomous and ordinal requiring ordinal regression to examine the relationship the independent variables have on these dependent variables. Polr from the MASS package (with hess = true) was used to conduct the ordinal regression analysis (Venables and Ripley 2002). Logistic regression was used (glm with family = binomial) to examine their interest in becoming certified to recommend marijuana (R Core Team 2018).

Variables were grouped based on their category (physicians’ beliefs about medical marijuana [7 variables], concerns about medical marijuana [6 variables], and perceived knowledge and ability [4 variables]). The first set of analyses entered all the variables within each category alone. The first regression analysis involved physician beliefs about medical marijuana and the seven associated variables were used for this regression analysis. The second analysis utilized concerns about medical marijuana and its six variables, and the third analysis used perceived knowledge and ability and its four variables. The final analysis utilized a stepwise regression (method = both, adds and drops variables in order to attain best fit) with all seventeen variables entered at once (Chambers and Hastie 1992). The model fit was measured using the Akaike’s information criterion (AIC), where a lower number indicates a more parsimonious model compared to models with a higher AIC.

## Results

As shown in Table 1, the sample population was predominately heterosexual, white, and male. Table 2 highlights how the sample was not supportive of Ohio’s program with most saying that it was too lenient. Most physicians had no plans to become certified to recommend marijuana within the state, and most were not likely to recommend marijuana to their patients. Physicians were not generally supportive of medical marijuana and did not believe in its safety or its legality.

**Table 1:** Demographic characteristics of 348 Ohio physicians from an internet-based survey

	Percent	n
**Cisgender Male**	70.8%	237
**Heterosexual**	92.8%	332
**Race/Ethnicity**		
White	87.6%	304
Asian-Indian	3.8%	13
Black	1.2%	4
Native American/ Hawaiian/Pacific Islander	2.0%	3
Other Asian	1.4%	5
Other race	4.0%	14
**Hispanic**	2.5%	8

**Table 2:** Beliefs and attitudes towards medical cannabis among 348 Ohio physicians from an internet-based survey

	Percent	n	
**Likely to Recommend**			
Extremely or Somewhat Unlikely	62%	215	
Neither Likely Nor Unlikely	13%	45	
Extremely or Somewhat Likely	25%	87	
**Opinion about Program**			
Too Lenient	41.2%	127	
Too Strict	24.7%	76	
Just Right	34.1%	105	
**Interest in Certification**			
Already Certified or Plans for Certification	12.2%	42	
No Plans for Certification	87.8%	303	
**Beliefs About Medical Marijuana**^**a**^	**Mean**	**Stand Dev**	**n**
Medical marijuana should be legal	2.73	1.50	346
Medical marijuana is safe when used responsibly	2.61	1.38	344
Marijuana has medicinal uses	2.18	1.16	348
Medical marijuana will increase crime rates	3.61	1.27	344
Medical marijuana will hurt the war on drugs	3.46	1.41	347
Those supporting medical marijuana are primarily interested in recreational use	2.36	1.25	347
Marijuana is harmful to people	2.48	1.13	348
**Concerns About Marijuana**^**b**^			
Safety	2.51	0.98	347
Consistency of quality	2.80	1.02	348
Concern about the federal law	2.63	1.05	346
Concern about psychoactive effects	2.73	1.03	347
Limited evidence of therapeutic benefits	2.70	1.08	348
Potential addiction to marijuana	2.49	1.06	344
**Perceived Knowledge About Medical Marijuana**^**a**^			
Knowledgeable about medical marijuana	2.78	1.20	346
Answering patient questions about efficacy	2.85	1.30	346
Answering patient questions about safety	2.57	1.21	345
Answering patient questions about drug interactions	3.15	1.32	345

a. Strongly agree, Somewhat agree, neither agree nor disagree, somewhat disagree, strongly disagree

b. Not Concerned, somewhat concerned, moderately concerned, extremely concerned

The data in Table 3 examines the relationship between beliefs, concerns, and perceived knowledge of the physician’s likelihood of recommending marijuana, their intention to become certified, and their opinion about Ohio’s program. The likelihood of physicians recommending marijuana to their patients was influenced by only a few factors. Overall, physician opinions and concerns supporting cannabis use were related to their likelihood to recommend, to become certified, and to feel that Ohio’s program was too strict. None of the perceived knowledge questions were significantly related to any of the dependent variables on their own.

**Table 3:** Attitudinal factors associated with physicians beliefs, concerns, and perceived knowledge regarding Ohio’s medical marijuana program

	Likely To Recommend ***n*** = 314		Intention To Be Certified n = 314		Opinion toward Ohio’s Program ***n*** = 274	
Beliefs About Medical Marijuana *Strongly Agree-Strongly Disagree*	OR	CI 2.5%/97.5%	OR	CI 2.5%/97.5%	OR	CI 2.5%/97.5%
Medical marijuana should be legal	.43	.26 / .67 ***	.32	.12 / .72 *	.42	.28/ .63***
Medical marijuana is safe when used responsibly	.90	.56 / 1.44	.84	.34 / 1.87	.82	.56/ 1.20
Marijuana has medicinal uses	.48	.28 / .78 **	.99	.45 / 2.04	1.05	.71/ 1.54
Medical marijuana will increase crime rates	1.19	.81 / 1.74	1.56	.86 / 3.04	1.25	.90/ 1.75
Medical marijuana will hurt the war on drugs	1.13	.80 / 1.59	.67	.41 / 1.17	1.26	.93/ 1.70
Those supporting medical marijuana are primarily interested in recreational use	1.12	.87 / 1.45	.99	.70 / 1.40	.96	.75/ 1.23
Marijuana is harmful to people	1.26	.93 / 1.71	1.27	.86 / 1.90	1.50	1.12/ 2.00**
	AIC = 410.74**		AIC = 197.65***		AIC = 441.86***	
**Concerns About Marijuana***Not Concerned To Extremely Concerned*						
Safety	.72	.48 / 1.08	.44	.24 / .80 **	.61	.42/ .90**
Consistency of quality	1.48	1.08 / 2.04 *	1.59	1.04 / 2.46 *	1.46	1.07/ 2.00*
Concern about the federal law	1.04	.78 / 1.38	1.17	.79 / 1.74	1.31	1.00/ 1.71*
Concern about psychoactive effect	.52	.35 / .79 **	.82	.46 / 1.44	.60	.40/ .88**
Limited evidence of therapeutic benefits	.39	.28 / .53 ***	.44	.27 / .69 ***	.50	.37/ .67***
Potential addiction to marijuana	.88	.61 / 1.26	1.08	.63 / 1.88	.57	.40/ .80***
	AIC = 480.69***		AIC = 216.45***		AIC = 490.76***	
**Perceived Knowledge About Medical Marijuana***Strongly Agree-Strongly Disagree*						
Knowledgeable about medical marijuana	.83	.61 / 1.12	.58	.32 / .99	.89	.67/ 1.18
Answering patient questions about efficacy	1.07	.75 / 1.52	.57	.30 / 1.08	1.25	.90/ 1.74
Answering patient questions about safety	.93	.69 / 1.26	1.25	.68 / 2.31	.88	.66/ 1.17
Answering patient questions about drug interactions	.89	.70 / 1.12	1.08	.76 / 1.50	.96	.77/ 1.21
	AIC = 626.01		AIC = 237.47***		AIC = 667.00	

*P*-Value * < = .05 *P*-Value ** < = .01 P-Value *** < = .001

OR = odds ratios, 95% CI = confidence interval, AIC = Akaike’s information criterion obtained from ordinal and logistic regressions

The variables with the greatest statistical relationship with physicians’ likelihood to recommend cannabis were the opinions that medical marijuana should be legal and that it has medicinal uses. Physicians were approximately twice as likely to recommend marijuana to their patients if they believed that medical marijuana should be legal. Believing that medical marijuana should be legal and that it has medicinal uses was associated with a greater likelihood of recommending marijuana to patients. Concerns about the lack of evidence and marijuana’s psychoactive effects were negatively related to their interest in recommending marijuana to patients.

The intention to become certified was affected mostly by concerns about marijuana. Physicians were less likely to become certified if they felt that there was limited evidence toward its use and if they were concerned about its quality. However, having concerns about its quality meant that physicians were more likely to become certified. Other than their concerns, physician belief that medical cannabis should be legal was associated with the intention to become certified.

Greater concern about marijuana’s safety, limited evidence, potential addiction, and its psychoactive effects were associated with physicians believing that the program is too lenient. By contrast, concerns about the consistency of quality and the federal laws were associated with the belief that the program was too strict. Regarding the physician opinions, a greater belief that medical marijuana should be legal was associated with the opinion that Ohio’s program is too strict, whereas, believing that marijuana is harmful was associated with the belief that Ohio’s program is too lenient.

Table 4 presents the results of the stepwise regressions containing all seventeen variables.

**Table 4:** Stepwise selected attitudinal factors associated with physicians beliefs, concerns, and perceived knowledge regarding Ohio’s medical marijuana program

	Likely To Recommend n = 314		Intention To Be Certified n = 314		Opinion toward Ohio’s Program n = 274	
	OR	CI 2.5%/97.5%	OR	CI 2.5%/97.5%	OR	CI 2.5%/97.5%
Medical marijuana should be legal	.37	.24 / .54 ***				
Marijuana has medicinal uses	.48	.29 / .78 **				
Medical marijuana will hurt the war on drugs	1.25	.93 / 1.69				
Those supporting medical marijuana are primarily interested in recreational use	1.25	.96 / 1.61				
Knowledgeable about medical marijuana	.77	.56 / 1.05				
Answering patient questions about drug interactions	.73	.54 / .98 *				
	AIC = 379.32**					
Medical marijuana should be legal			.21	.10 / .38 ***		
Concern about safety			.50	.25 / .95 *		
Concern about consistency of quality			1.48	.94 / 2.39		
Concern about potential addiction to marijuana			2.01	1.12 / 3.80 *		
Knowledgeable about medical marijuana			.32	.14 / .66 **		
Answering patient questions about efficacy			.41	.17 / .90 *		
Answering patient questions about safety			2.41	1.08 / 6.09 *		
			AIC = 157.5***			
Medical marijuana should be legal					.39	.30/ .51***
Medical marijuana will hurt the war on drugs					1.43	1.10/ 1.85**
Concern about safety					.67	.46/ .95*
Concern about the federal law					1.38	1.04/ 1.83*
Potential addiction to marijuana					.64	.45/ .90**
					AIC = 414.26**	

*P*-Value * < = .05 *P*-Value ** < = .01 *P*-Value *** < = .001

*OR* Odds ratios, 95% CI = confidence interval, AIC = Akaike’s information criterion obtained from ordinal and logistic regressions utilizing stepwise deletion

The physicians were more likely to recommend medical marijuana to their patients if they believed that medical marijuana should be legal and that it has medicinal uses. Furthermore, greater ability to answer patient questions about drug interactions with marijuana was associated with being less likely to recommend marijuana to their patients.

The intentions to become certified, was affected by a physician’s support for the legalization of medical marijuana, where greater support for legalization indicated greater intention of becoming certified. Conversely, greater perceived knowledge about medical marijuana was associated with a lower intention of becoming certified. Concerns about safety and ability to answer questions about efficacy were associated with a lower intention of becoming certified. Greater concern about addiction and ability to answer questions about safety was associated with a greater intention of becoming certified.

The opinion of physicians regarding Ohio’s program was most affected by their belief that medical marijuana should be legal. Those who felt that medical marijuana should be legal generally felt that Ohio’s program is too strict. Concern about the potential addiction to marijuana and its safety were associated with the opinion that the program is too lenient. Further, a belief that medical marijuana hurts the war on drugs and concerns about federal marijuana laws were associated with the opinion that Ohio’s program is too strict.

## Discussion

This study examined Ohio physicians’ likelihood of recommending cannabis to their patients, their interest in becoming certified to recommend cannabis, and whether they believed Ohio’s program to be too strict or too lenient. The results indicate that these were primarily affected by their belief that medical marijuana should be legal. No other variables were consistently identified for each outcome as being significant. Some of the results appeared to be counter-intuitive. For example, greater concerns about potential addiction to marijuana and marijuana consistency were associated with a greater intention to become certified. Similarly, the belief that medical marijuana will hurt the war on drugs and a greater concern about federal law were both associated with a belief that Ohio’s program is too strict.

The contrary results could be due to problems with the validity of the measures used within the study. Specifically, that question may not be measuring what researchers expect. For example, believing that medical cannabis will hurt the war on drugs could be seen by people as a positive or negative outcome. Those that believe it to be a positive outcome could also believe that Ohio’s program is too strict. Having concerns about quality may not be a factor if physicians believe in the effectiveness of the program to control quality. These findings indicate elements that require further investigation, specifically with qualitative methods in order to come to a better understanding of physician attitudes and beliefs.

A study conducted by the State Medical Board of Ohio asked physicians how likely they were to recommend marijuana; approximately 40% stated that they were unlikely and 30% were likely to recommend marijuana (State Medical Board of Ohio 2016). The data within the current study similarly found that most physicians were unlikely to recommend marijuana to their patients. The decision to recommend marijuana or become certified was primarily associated with a physician’s belief about medical marijuana legalization rather than more specific concerns or beliefs. Moreover, the physicians’ concerns and perceived knowledge did not have as consistent or strong of a relationship compared to their belief about legalization.

### Policy considerations

The lack of interest in recommending or in becoming certified to recommend cannabis may explain the number of physicians who are currently certified to recommend cannabis in the state. as of October 2019, the roster identified 575 physicians who have been certified to recommend cannabis in the state of Ohio (Ohio Medical Marijuana Control Program 2019). There are also 63,819 registered patients in the state (who must visit a certified physician as part of the registration process); of those, 40,571 had purchased medical cannabis. It is unknown whether there are patients who want to become registered but cannot find a certified physician. The issue may not be just about numbers, but about location. Examining the map of certified physicians within the state shows clusters around Ohio’s major cities with gaps in more rural areas (State Medical Board of Ohio, 2019).

While the attitudes of the general public are increasingly becoming more positive toward cannabis, physicians’ attitudes are not changing in the same degree (Carliner et al. 2017). This disconnection between physicians and potential patients could lead patients to access cannabis (legal or not) without their physicians’ knowledge. A study conducted in New York found that many patients utilized one physician who was registered to recommend cannabis and another who was not for other care (Sideris et al. 2018).

Additionally, cannabidiol (CBD) has become increasingly common as an over the counter product in Ohio and in other jurisdictions where recreational cannabis remains illegal. It is unclear how access to over the counter CBD products will impact patients medical care, but one study found that many people use CBD for general health reasons and that it was recommended by a nonmedical practitioner (Corroon and Phillips 2018). In an earlier study, the same author found that people used cannabis as a replacement for prescription drugs (regardless of the legality in their state) (Corroon et al. 2017). This could be a problem, as another study found that primary care physicians may not know of their patients’ use of cannabis, and they found situations where a patient’s conditions could be affected by cannabis use (Kondrad et al. 2018). The implication is that many physicians will be providing care to patients who are using cannabis and cannabis products without the physician’s knowledge.

Ohio and other states are creating a parallel health system -- one for cannabis and another for traditional medicine -- and these systems are unconnected. As a result, patients need to have multiple appointments with physicians, potentially increasing the opportunity for mistakes, as the different physicians may not have a complete picture about their patient’s health. While it is important for physicians to be knowledgeable about cannabis in order to effectively recommend it, it is a mistake to assume that other physicians do not need this information. The growing interest in medical cannabis and the possibility for recreational use means that all physicians should be provided with information about the impact of cannabis on their patients.

Most physicians do not receive any medical school training about the use of cannabis in healthcare (Evanoff et al. 2017). It is possible that physicians who believe that medical cannabis should be legal are more likely to seek out education and information on the use of cannabis (in the case of these data, to become certified). The result could be like an echo chamber where physicians who are already biased toward the use of cannabis seek out and receive training, while those who may be more critical do not. This could create a problem where patients may seek care from different physicians, who may or may not know about each other. While medical cannabis users in Ohio are required to enroll in a registry, the same cannot be said for those using over the counter CBD products.

### Limitations

The sample in this study was not representative of all physicians in Ohio. Further, the 2.9% response rate could indicate a bias toward physicians who have strong opinions about the legality of medical cannabis. Both this study and the 2016 Ohio Board of Physicians study involved convenience samples with very low response rates. This means a high likelihood of sample bias confounding the results. Because the surveys were anonymous, we do not have data available to evaluate the degree to which survey respondents were representative of the entire population of Ohio physicians. Future studies using probability sampling techniques are needed to address this issue.”

In conclusion, little is known about the demographics of the physicians in Ohio; therefore, it is difficult to assess how different the study sample was from the larger population of physicians. These data lack enough diversity (e.g., race/ethnicity, gender, sexual orientation) to assess the impact of these factors on peoples’ attitudes toward cannabis. While the study asked about the physicians’ ability to provide patients with information about cannabis, it did not ask whether they received or sought out any information to educate themselves about cannabis, nor did the study conduct an objective test of physician knowledge. Further, the analysis focused on attitudes rather than actual behavior. Physicians may be supportive but may experience other barriers to becoming certified such as pressure from their practice. Even with these limitations, the trend that most physicians have concerns about the use of cannabis for medical problems is mirrored by other studies within and outside the state of Ohio.

## Data Availability

The data used for this study are available from the corresponding author with a reasonable request.
